# Mechanisms of Surround Suppression Effect on the Contrast Sensitivity of V1 Neurons in Cats

**DOI:** 10.1155/2022/5677655

**Published:** 2022-03-08

**Authors:** Hao Yu, Fei Xu, Xiangmei Hu, Yanni Tu, Qiuyu Zhang, Zheng Ye, Tianmiao Hua

**Affiliations:** College of Life Sciences, Anhui Normal University, Wuhu, Anhui 241000, China

## Abstract

Surround suppression (SS) is a phenomenon that a neuron's response to visual stimuli within the classical receptive field (cRF) is suppressed by a concurrent stimulation in the surrounding receptive field (sRF) beyond the cRF. Studies show that SS affects neuronal response contrast sensitivity in the primary visual cortex (V1). However, the underlying mechanisms remain unclear. Here, we examined SS effect on the contrast sensitivity of cats' V1 neurons with different preferred SFs using external noise-masked visual stimuli and perceptual template model (PTM) analysis at the system level. The contrast sensitivity was evaluated by the inverted threshold contrast of neurons in response to circular gratings of different contrasts in the cRF with or without an annular grating in the sRF. Our results showed that SS significantly reduced the contrast sensitivity of cats' V1 neurons. The SS-induced reduction of contrast sensitivity was not correlated with SS strength but was dependent on neuron's preferred SF, with a larger reduction for neurons with low preferred SFs than those with high preferred SFs. PTM analysis of threshold versus external noise contrast (TvC) functions indicated that SS decreased contrast sensitivity by increasing both the internal additive noise and impact of external noise for neurons with low preferred SFs, but improving only internal additive noise for neurons with high preferred SFs. Furthermore, the SS effect on the contrast-response function of low- and high-SF neurons also exhibited different mechanisms in contrast gain and response gain. Collectively, these results suggest that the mechanisms of SS effect on neuronal contrast sensitivity may depend on neuronal populations with different SFs.

## 1. Introduction

The response of a neuron to visual stimulation in the classical receptive field (cRF) is generally suppressed by a costimulation in the surrounding receptive field (sRF) beyond the cRF [1, 2]. This phenomenon, called surround suppression (SS), is widely reported at various levels along the visual pathway in many species [3] and thus commonly regarded as a fundamental property of visual neurons for efficient information processing [4, 5]. A neuron's response to dissimilar stimuli presented simultaneously in the cRF and sRF is usually stronger than that to similar stimuli [6, 7], and it is commonly thought that SS plays a critical role in the perception of visual saliency, detection of object boundaries, and figure-ground segregation [3, 7–12]. However, the neural mechanisms that SS mediates visual information processing have not been fully understood [3, 13–19].

Contrast detection is fundamental to visual perception of object form, size, and motion features [20, 21]. A few studies report that SS significantly reduces both the perceptual contrast sensitivity [22] and response contrast sensitivity of neurons in the primary visual cortex (V1) [2, 23]. Nevertheless, the neural mechanisms remain controversial [2, 23]. It is known that the perceptual contrast sensitivity exhibits an evident dependence on stimulus spatial frequencies (SFs) [24–28], and neuronal response contrast sensitivity to visual stimuli is also dependent on the preferred SFs of cortical neurons [28–32]. Furthermore, a recent report showed that SS strength has a positive correlation with the preferred SFs of V1 neurons [33]. These results lead to a hypothesis that the effect of SS on neuronal contrast sensitivity may depend on neuronal SFs. One goal of this study is to examine this possibility.

On the other hand, a majority of previous electrophysiological investigations focused on the SS effect on the response contrast sensitivity at the cellular level. Based on the contrast-response function fitting of individual neurons, some of these studies have reported inconsistent results [2, 23, 34, 35], probably because SS effects on contrast encoding might not only operate at the cellular level, but also involve a complex interaction between feed-forward, lateral, and feedback connections [3, 36, 37]. Conversely, studies on perceptual contrast sensitivity functions are widely conducted at the system level to model observer performance in perceptual tasks using the perceptual template model (PTM) in terms of perceptual template(s), transducer nonlinearity, internal additive noise, and multiplicative noise [24, 38–40]. This PTM has been used to distinguish mechanisms of attention [41–43], perceptual learning [44–47], and top-down [48] effects on threshold contrast in the detection of visual signals. To identify the mechanisms of SS effects on neuronal contrast sensitivity at the system level, the present study applied the external noise paradigm and the PTM analysis to measure the SS effect on the contrast sensitivity of V1 neurons with different preferred SFs. The contrast sensitivity was assessed with the inverse of signal threshold contrast that V1 neurons could respond to visual stimuli masked by gradient levels of external noise using receiver operating characteristics (ROC) analysis with different performance accuracies (70.7% and 79.4%) [49, 50]. The signal threshold versus external noise contrast (TvC) functions were constructed for neuronal populations with different spatial frequencies (SFs). Based on PTM analysis of neuronal TvC functions with and without surround stimuli, we try to distinguish the mechanisms underlying SS effect on neuronal contrast sensitivity. Finally, we also assessed the SS effect on neuronal contrast-response functions to examine the relationship of SS effect between system and cellular level.

## 2. Materials and Methods

### 2.1. Animals and Preparation

Four healthy young adult cats (2-3 years old, weighing 2.4-3.1 kg) were used in this study. All cats were examined with an ophthalmoscope before the experiment to ensure that they had no optical or retinal abnormality. All animal treatments were performed strictly in accordance with the Guide for the Care and Use of Laboratory Animals of the National Institutes of Health. The experiment protocol in this study was approved by the Animal Welfare Ethics Committee of Anhui Normal University.

Animal anesthesia and preparation were carried out using the same procedure as described previously [51–54]. Briefly, anesthesia was initiated by injection of ketamine HCl (40 mg/kg, i.m.) and xylazine (2 mg/kg, i.m.). After intubation of intravenous and tracheal cannulae, the cat was immobilized in a stereotaxic apparatus with ear, eye, and bite bars. Glucose (5%)-saline (0.9%) solution containing a mixture of urethane (20 mg/h/kg) and gallaminetriethiodide (10 mg/h per kg of body weight) was infused intravenously by a syringe pump to keep the animal anesthetized and paralyzed. Pupils were dilated with atropine (1%) eye drops, and contact lenses (zero power) were used to protect the corneas from dryness. Neosynephrine (5%) was applied to retract the nictitating membranes. Artificial respiration was performed, and expired pCO_2_ was maintained at approximately 3.8%. Body temperature was maintained at 38°C using a heating blanket. The animal's electrocardiogram, heart rate (180-220 beats/min), and blood oxygen level (>95%) were monitored continuously throughout the experiment to evaluate the anesthesia level and physiological state.

### 2.2. Electrophysiological Recording

The V1 area (area 17) (Horsley-Clarke coordinates: P0-P8/L0-L4) was exposed by performing a craniotomy on the skull under a surgery microscope, and dura over V1 was cut and removed. The exposed V1 area was covered with a 4% agar. Extracellular single-unit recordings were carried out using a glass-coated microelectrode (impedance 3-5 M*Ω*), which was advanced by a hydraulic micromanipulator (Narishige, Japan). The optic discs of the two eyes were reflected onto a movable transparent tangent screen positioned 57 cm away from the eyes and overlapped with the CRT monitor for visual stimuli presentation. The central areas of both eyes were located as previously described [32, 51, 52, 55]. V1 neurons were randomly sampled from all cortical layers in the medial bank of the lateral gyrus with the electrode penetrations within a vertical depth of 2000 *μ*m from the pial surface. Action potentials of the recorded neurons were amplified (×2,000) by a microelectrode amplifier (Dagan 2400A, USA) and then fed into a window discriminator with an audio monitor. The original voltage traces were digitized by an acquisition board (National Instruments, USA) controlled by IGOR software (Igor Pro 6.3.1.2, WaveMetrics, USA) and then saved for online and offline analysis.

### 2.3. Visual Stimuli and Recording Procedure

Visual stimuli were drifting sinusoidal gratings or noise gratings generated and displayed in real time by a PC computer running Matlab programs (Matlab 2014a, MathWorks, USA) with the aid of Psychtoolbox extensions [56]. The grating and external noise images had a fixed mean luminance of ~19 cd/m^2^ and were presented on a CRT monitor (Legend LXH-GJ769FT, China) with a resolution of 1024 × 768 pixels and a refresh rate of 60 Hz. The luminance nonlinearity of the monitor was gamma corrected. The monitor was placed 57 cm from the cat's eyes.

Once a neuron's visually evoked response was detected, the neuron's RF center was preliminarily determined using bars of light emitted from a hand pantoscope and then precisely located by consecutively presenting a series of computer-generated flickering squares of light on the CRT. The cell's preferred orientation, motion direction, spatial, and temporal frequency were determined by comparing the cell's responses to a series of grating stimulus packages and used for subsequent experiments.

**Figure 1:**
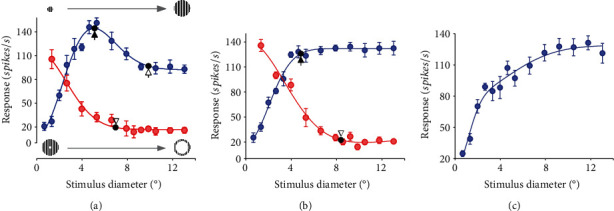
Samples of response tuning curves showing RF property of different types of V1 neurons. (a) Size-tuning responses to circular patch grating stimuli (upper left to right corner) with increasing outer diameter (blue solid circles with error bars, *n* = 56) and to annular grating stimuli (lower left to right corner) with increasing inner diameter (red solid circles with error bars, *n* = 48) for neurons with evident surround suppression (SS). The blue and red solid curves are the best fits of size-tuning responses with DoG functions. (b) Size-tuning responses to circular patch grating stimuli with increasing outer diameter (blue color) and to annular grating stimuli with increasing inner diameter (red color) for neurons with no surround suppression (NOS). (c) Size-tuning responses to circular patch grating stimuli with increasing outer diameter for neurons showing facilitated response to sRF stimulation. Solid arrows indicate the diameter of cRF corresponding to the maximum response (in (a)) or 95% of the saturation response (in (b)), and the open arrow defines the outer boundary of sRF in the size-tuning response to circular patch grating stimuli. Arrowheads indicate the nonresponsive area in the size-tuning response to annular grating stimuli.

To explore the surround suppression effect on the contrast sensitivity of the studied neuron, we characterized neuron's RF properties, including the size of cRF and sRF. We first recorded the neuronal response to circular patch drifting gratings with different sizes of diameter centered over the RF center (Figure 1). Four repetitions of recording were carried out. The response was measured as a function of different stimulus patch diameters. By performing an online fit of each recorded cell's size-tuning curves with a DoG function [57], neurons were classified into three categories based on their size-tuning curves. The first type of neurons, characterized by an evident response suppression after reaching a maximum response (Figure 1(a)), were named surround-suppressive neurons (NSS). The second type of neurons displayed an asymptotic maximum response without surround suppression and were called nonsurround-suppressive neurons (NOS) (Figure 1(b)). The third type of neurons showed facilitated responses as the size of stimulus increased and therefore were named surround-facilitative neurons (NSF) (Figure 1(c)). For NSS neurons, the diameter of cRF was defined as the circular-patch size corresponding to the maximum response, and the diameter of sRF outer boundary was defined as the size corresponding to an asymptotic response value (the minimum response plus 5% of the minimum response) (Figure 1(a)). For NOS neurons, the diameter of cRF was defined as the circular-patch size corresponding to 95% of the saturation response (Figure 1(b)). There was no suppressive sRF beyond the cRF for NOS neurons. NSF neurons had unmeasurable cRF (Figure 1(c)).

The inner boundary of sRF for NSS and NOS neurons was determined by recording the neuron's response to a series of annular drifting gratings centered over the RF center, with the annulus's inner diameter expanding and the outer diameter fixed at the maximum size available on the display (Figure 1(a)). After fitting the annulus size-tuning curves with a DoG function [57], the inner diameter of sRF was defined as the annulus size corresponding to the response value of minimum response (spontaneous response) plus 5% of the minimum response in the fitting curve (Figures 1(a) and 1(b)). The inner diameter of sRF was measured in order to ensure that an annular surround stimulus beyond this boundary did not drive the neuron's visually evoked response.

**Figure 2:**
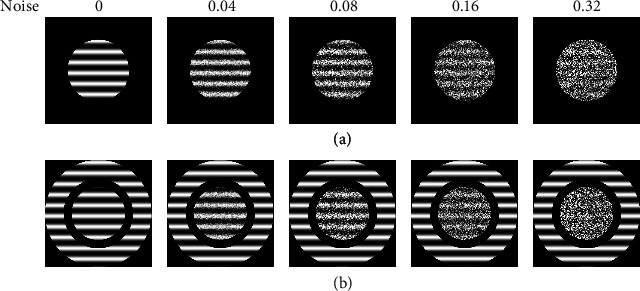
Showing circular patch grating stimuli with different external noise levels (0, 0.04, 0.08, 0.16, and 0.32) presented only in the cRF (a) as well as noisy circular patch grating stimuli plus annular grating stimuli presented in the sRF (b). The circular grating stimuli and the annular grating stimuli had the same orientation, motion direction, spatial, and temporal frequency as the neuron's preferred parameters. The diameter of circular grating stimuli was equal to the neuron's cRF. The outer diameter of annular grating stimuli was set as large as our display size, and the inner diameter was set equal to the size corresponding to the asymptotic response on the annular size-response tuning curve. The contrast of circular grating stimuli varied between 0% and 100%, and the contrast of annular grating stimuli was fixed at 100%.

In order to examine surround suppression effects on the contrast sensitivity of NSS neurons, we recorded neuronal responses to circular-patch noise grating stimuli with gradient luminance contrast (0, 0.025, 0.05 0.1, 0.2, 0.3, 0.4, 0.5, 0.6, 0.8, and 1.0) presented in the cRF when the annular grating stimuli were present or absent in the sRF (Figures 2(a) and 2(b)). As controls, the contrast sensitivity of NOS neurons to circular-patch noise grating stimuli in the cRF was also examined when the annular grating stimuli were shown or absent in the sRF. The noise gratings in the cRF were composed of external noise frames and signal frames. The signal frames were drifting gratings with the neuron's preferred orientation, spatial frequency, and temporal frequency. The external noise frames had the same size as that of the signal frames with each noise element subtending 2 × 2 pixels. The gray levels of the noise elements in each external noise frame were drawn independently from a Gaussian distribution with mean 0 and standard deviation depending on the amount of external noise for each noise condition. To ensure that the external noise did conform to the Gaussian distribution, the maximum standard deviation of the noise was kept below 33% maximum achievable contrast [24, 40, 44, 58, 59]. Five external noise levels (0.0, 0.04, 0.08, 0.16, and 0.32) were used in this experiment. The noise grating with different contrasts at different external noise levels were presented in a random order and repeated for 6 sessions (5 trials/session) when the annular gratings were present or absent. To maximize and fix the surround suppression influence, the annular grating stimuli presented in the sRF were free of external noise mask, maintained at 100% contrast, and had the same orientation, spatial frequency, and temporal frequency as the circular patch noise gratings shown in the cRF. Prior to presentation of each trial of stimulus, spontaneous activity was acquired during 1 s period while the mean luminance was shown on the CRT. At the end of the recording experiment, animals were killed by stopping its heart beat and breath through intravenous injection of pentobarbital sodium (>100 mg kg^−1^).

### 2.4. Data Analysis

#### 2.4.1. Size-Tuning Curve Fitting and Surround Suppression Index

Neuron's responses to circular and/or annular grating stimulus were defined as the mean firing rate corresponding to the time of stimulus presentation. The size-tuning curve of neuronal response to the circular patch or annular grating stimuli was fitted with a DoG function [57]:

(1) $$\begin{array}{c} R\left(x\right)=K_{c}\times \overset{x/2}{\underset{-x/2}{\sum }}\exp{\left(-\left(2\times x/r_{c}\right)\right)}^{2}-K_{s}\times \overset{x/2}{\underset{-x/2}{\sum }}\exp{\left(-\left(2\times x/r_{s}\right)\right)}^{2}+R_{0}, \end{array}$$

where *R*(*x*) is the response evoked by a circular patch grating stimulus with diameter *x*, *K*_*c*_ is amplitude of the center subunit, *r*_*c*_ is the radius of the center subunit, *K*_*s*_ is the amplitude of the surround subunit, and *r*_*s*_ is the radius of the surround subunit. *R*_0_ is the spontaneous firing rate. The surround subunit radius was taken to be the spatial extent of the sRF.

The strength of the surround suppression was assessed using the surround suppression index (SI) defined by the following equation:

(2) $$\begin{array}{c} \text{SI}=1-\frac{\text{Asymptotic response}}{\text{Peak response}}, \end{array}$$

where the peak response is the neuron's maximum response to circular patch grating stimuli with increasing diameter, and the asymptotic response indicates a response value of the saturation response plus 5% saturation response in the size-response tuning curve (Figure 1(a)). The larger the SI, the stronger the surround suppression.

#### 2.4.2. ROC Analysis

**Figure 3:**
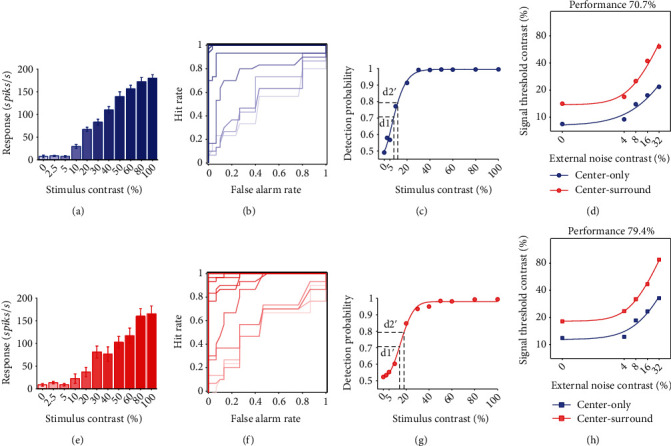
A sample cell showing the ROC analysis for measurement of the contrast threshold that the V1 neuron can respond to the visual stimuli at two performance criteria (d1′ = 70.7% and d2′ = 79.4%) with and without the presence of surround stimuli. (a, e) Contrast response functions showing the mean neuronal response to repeated presentation (6 × 5 trials) of visual stimuli with different luminance contrast (0, 0.025, 0.05, 0.1, 0.2, 0.3, 0.4, 0.5, 0.6, 0.8, and 1.0, as indicated by the color gradient) at an external noise level of 0 without (a) and with (e) surround stimuli. (b, f) ROC method shown by the hit rate versus false alarm rate for neuronal response value distribution at different stimulus contrasts compared with that of baseline response without (b) and with (f) surround stimuli. (c, g) showing the probability of the neuron in detection of visual stimuli with different contrasts without (c) and with (g) surround stimuli. The solid curves represent the best fits of detection probability versus stimulus contrast functions with a Weibull equation. The dashed lines indicate the signal contrast threshold (TC) at two performance criterion of d1′ and d2′, respectively. (d, h) TC versus external noise contrast (TvC) functions at performance criterion 70.7% (d) and 79.4% (h).

The correct performance of a neuron's response to a certain stimulus contrasts at several external noise levels with and without surround stimuli was assessed using receiver operating characteristics (ROC) analysis [32, 49, 50]. Briefly, the neuronal detection probability was computed by designating the number of spikes elicited by a certain stimulus contrasts (0, 0.025, 0.05, 0.1, 0.2, 0.3, 0.4, 0.5, 0.6, 0.8, and 1.0) as “hit” and spontaneous firing rate as “false alarm” (Figure 3). After fitting the detection probability versus stimulus contrast with a Weibull function, we obtained the neuronal threshold contrast (TC) in response to visual stimuli at a certain external noise level under 70.7% (d1′ = 1.089) and 79.4% (d2′ = 1.634) detection accuracy, which was used to construct TC versus external noise contrast (TvC) functions with and without surround stimuli at different performance criteria. The neuronal contrast sensitivity (CS) was evaluated by the inverse of TC value to construct contrast sensitivity versus spatial frequency (CSF) functions.

#### 2.4.3. PTM Modeling Analysis

**Figure 4:**
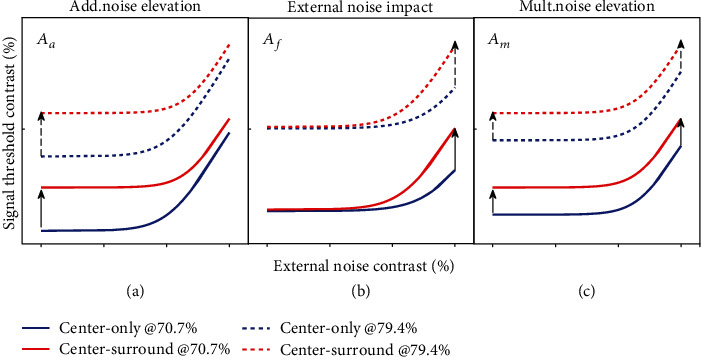
Possible mechanisms of surround suppression effect on neuronal contrast sensitivity predicted by PTM. Each panel shows how TvC curves at two performance criteria would change during surround suppression for changes in one of three PTM parameters where *A*_*a*_ represents internal additive noise (a), *A*_*f*_ represents external noise exclusion (b), and *A*_*m*_ represents multiplicative noise (c). In all three panels, blue curves represent TvCs from the center-only group, whereas red curves represent TvCs from the center-surround group. Solid lines represent less stringent performance criteria (70.7%); dotted lines represent more stringent performance criteria (79.4%). Arrows represent the hypothetical size and direction of change in performance affected by surround suppression.

The perceptual template model (PTM) considers the observer as a whole system and describes the observer's performance in terms of a perceptual template with a tuning gain of stimulus representation (*β*), a nonlinear signal transducer function (*γ*), an internal multiplicative noise (*N*_*m*_) whose standard deviation is proportional to stimulus contrast (or signal strength), and an internal additive noise (*N*_*a*_) whose amplitude is independent of stimulus contrast [41, 44, 60–62]. Equivalent internal noise can be estimated by comparison to the effects of external noise (*N*_ext_) added in the stimulus. By measuring the observer's performance in the detection of visual signals with different amounts of external noise, the PTM analysis can identify three pure mechanisms of (1) stimulus enhancement (equivalent to internal additive noise reduction), (2) external noise exclusion and (3) internal multiplicative noise suppression (Figure 4), or a mixture of these mechanisms in attention [38, 42], perceptual learning [44–46, 58], top-down influence [48], and brain development [63]. To assess the relative contributions from each or combinations of the noise sources to SS effect on neuronal contrast sensitivity, three weighting coefficients *A*_*a*_, *A*_*f*_ , and *A*_*m*_ corresponding to the noise source *N*_*a*_, *N*_ext_, and *N*_*m*_, respectively, were added in the original PTM equation to fit neuronal TvC functions obtained with and without surround stimuli:

(3) $$\begin{array}{c} c_{\tau }=\frac{1}{\beta }{\left[\frac{\left(1+{\left(A_{m}N_{m}\right)}^{2}\right){\left(A_{f}N_{\mathrm{ext}}\right)}^{2\gamma }+\left.{\left(A_{a}N_{a}\right)}^{2}\right)}{\left(1/d\prime^{2}-N_{m}^{2}\right)}\right]}^{\frac{1}{2\gamma }}, \end{array}$$

where *C*_*τ*_ represents threshold contrast at the *d*′ performance level; *N*_*a*_, *N*_ext_, *N*_*m*_, *β*, and *γ* denote, respectively, the standard deviation of internal additive noise, the standard deviation of external noise, the proportional constant of multiplicative noise, the gain of the perceptual template, and the exponent of the nonlinear transducer. *A*_*a*_, *A*_*f*_, and *A*_*m*_ are the weighting coefficients of *N*_*a*_, *N*_ext_, and *N*_*m*_, respectively.

A least square procedure was used to fit the PTM equation. The fit was performed in Matlab 2014a with the curvefit toolbox extension. The sum of the squared differences between the measured and model-predicted log thresholds was minimized. The goodness of fit was determined by:

(4) $$\begin{array}{c} r^{2}=1.0-\frac{\sum {\left[\log\left({C_{t}}^{\text{predict}}\right)-\log\left(C_{t}\right)\right]}^{2}}{\sum {\left\{\log\left(C_{t}\right)-\text{mean}\left[\log\left(C_{t}\right)\right]\right\}}^{2}}. \end{array}$$

*A*_*a*_, *A*_*f*_ , and *A*_*m*_ were free to change in the full model to incorporate all three mechanisms predicted by the PTM. A reduced model was constructed by setting at least one of the three coefficients to 1.0. An *F* statistic was used to compare the reduced models to the full model:

(5) $$\begin{array}{c} F\left({df}_{1},{df}_{2}\right)=\frac{\left(r_{\text{full}}^{2}-r_{\text{reduced}}^{2}\right)/{df}_{1}}{\left(1-r_{\text{full}}^{2}\right)/{df}_{2}}, \end{array}$$

where *df*_1_ = *k*_full_–*k*_reduced_ and *df*_2_ = *N*–*k*_full_; *N* is the number of predicted data points.

The standard deviation of each model parameter for the best-fitting model was estimated with a bootstrap resampling method as described previously [45, 47, 59, 63]. The iteration in the bootstrap procedure is 1000 times. Thus, the mean and standard deviation of the best-fitting model parameters were obtained.

#### 2.4.4. Contrast Response Model Fitting

To identify the mechanisms of SS effect at the cellular level, we fitted the contrast response function of each NSS neuron, respectively, with three models based on Michaelis-Menten equation, including the response gain (Eq. (6)), contrast gain (Eq. (7)), and response subtraction model (Eq. (8)) constructed in the previous study [2].

Response gain model is expressed as

(6) $$\begin{array}{c} R=K\left(c_{s}\right){\left(\frac{c_{c}}{\sqrt{\sigma +c_{c}^{2}}}\right)}^{\beta }+R0, \end{array}$$

where *R* is the neurons' response, *K* (*c*_*s*_) is the scaling factor dependent on surround contrast, *c*_*c*_ is the center contrast, *σ* sets the neurons' contrast gain, and *β* sets the slope of the neurons' contrast response function in log-linear coordinates.

The contrast gain model is expressed as

(7) $$\begin{array}{c} R=K{\left(\frac{c_{c}}{\sqrt{\sigma \left(c_{s}\right)+c_{c}^{2}}}\right)}^{\beta }, \end{array}$$

where the response-scaling factor *K* is fixed, but the contrast gain parameter *σ* (*c*_*s*_) depends on surround contrast. Other parameters are the same as in the response gain model.

The response subtraction model is expressed as

(8) $$\begin{array}{c} R=\max\left[0,K{\left(\frac{c_{c}}{\sqrt{\sigma +c_{c}^{2}}}\right)}^{\beta }-k_{0}\left(c_{s}\right)\right], \end{array}$$

in which *k*_0_ (*c*_*s*_) is a response offset that depends on surround contrast, and other parameters are the same as in the two previous models.

To evaluate which model best characterized a neuron's contrast response function, we computed the goodness of fit using a normalized *χ*^2^ method. The *χ*^2^ was computed by

(9) $$\begin{array}{c} \chi^{2}=\underset{i}{\sum }\frac{{\left(e_{i}-o_{i}\right)}^{2}}{\sigma_{i}^{2}}, \end{array}$$

where *i* is the index of a particular contrast level, *e* is the expected response at this contrast level, *o* is the observed response, and *σ* is the trialwise standard deviation in responses at this contrast. The *χ*^2^ error term is then normalized by the degrees of freedom of the model to obtain *χ*_*N*_^2^. The lowest *χ*_*N*_^2^ indicates the best fitting and most efficient model [2].

All values were expressed as mean ± SD. The CSF and TvC functions with and without surround stimuli were compared using ANOVA and post-hoc test. All fitting was performed in MATLAB.

## 3. Results

### 3.1. Analysis of RF Properties and Surround Suppression Strength

A total of 93 V1 neurons from four cats were recorded and analyzed in this study. As shown by the fitting of DoG function to the circular grating size-response tuning curves, 61 neurons (NSS) exhibited an evident surround suppression, 24 neurons (NOS) had no surround suppression, and 8 neurons (NSF) displayed a facilitated response to expanding surround stimuli (Figure 1). This result was consistent with previous observations [2, 13, 64]. Based on the DoG function fitting, we, respectively, estimated neurons' cRF diameter corresponding to the maximum response (for NSS cells) or 95% of the saturation response (for NOS cells) as well as the diameter of sRF outer boundary corresponding to the asymptotic response in the size-tuning curves. The mean cRF diameters of NSS and NOS neurons were 5.83° ± 1.48° and 5.57° ± 1.32°, respectively. The mean sRF outer boundary diameter of NSS neurons was 12.28° ± 2.24°. Based on calculation of Eq. (2), the mean surround suppression index (SI) of NSS neurons was 0.21 ± 0.11 (Table 1).

Previous studies reported that there is a small region between the cRF outer boundary and sRF, where annular grating stimuli alone can elicit visually evoked response [2]. We also examined this responsive region for NSS and NOS by examining neuronal responses to annular grating stimuli with increasing inner diameters (Figure 1). Based on the DoG function fit to annular grating size-response tuning curves, our results showed that both NSS neurons and NOS neurons had this responsive region, with a mean minimum inner diameter of 8.73° ± 2.24° and 9.10° ± 1.00°, respectively (Table 1), which were similar to previous reports [2, 64].

### 3.2. Effects of Surround Suppression on Neuronal Contrast Sensitivity

**Figure 5:**
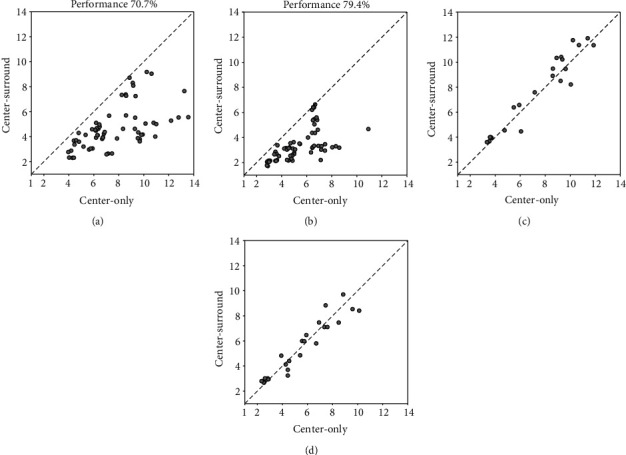
Scatter plots showing the neuronal contrast sensitivity value with surround stimuli (center-surround) versus without surround stimuli (center-only) at performance accuracy of 70.7% (a, c) and 79.4% (b, d) for neurons showing surround suppression effect (a, b) and no surround suppression effect (c, d).

To examine how surround suppression affected neuronal contrast sensitivity (CS) in response to visual stimuli in cRF, we first compared the CS of all studied neurons to visual stimuli without external noise mask in cRF with and without the presence of annular stimuli in sRF. As shown in the scatter plots, the CS value of most NSS neurons was reduced at both low and high performance criteria with surround stimuli relative to without surround stimuli (Figures 5(a) and 5(b)). Paired *t*-test indicated that the mean CS of NSS neurons with surround stimuli was significantly decreased at two performance criteria when compared with those without surround stimuli (*p* < 0.01 at both 70.7% and 79.4%). In contrast, the CS value of NOS neurons with and without the presence of surround stimuli was basically identical or quite close (Figures 5(c) and 5(d)). Paired *t*-test showed that the mean CS had no significant change at two performance criteria with surround stimuli versus without surround stimuli (*p* > 0.05 at both 70.7% and 79.4%).

**Figure 6:**
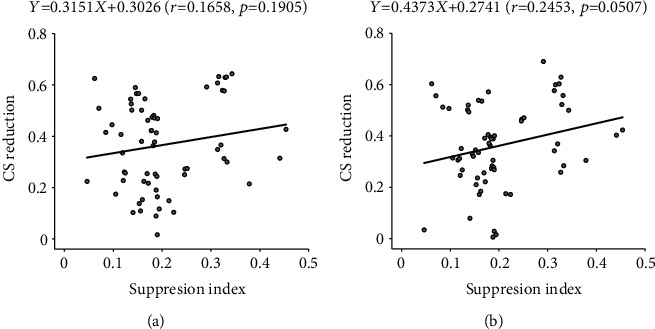
Scatter plots showing the correlation between the reduction amplitude (%) in the contrast sensitivity (CS) and surround suppression strength of all NSS neurons at performance criterion 70.7% (a) and 79.4% (b).

To explore if the surround suppression effect on neuronal CS depended on the strength of the surround suppression, we examined the correlation between the CS alterations and the surround suppression index (SI) of SS neurons. Our results showed that the amplitude of reduction in neuronal CS after surround suppression was not significantly correlated with the corresponding SI value at both performance criteria levels (70.7%: *r* = 0.1658, *p* = 0.1905; 79.4%: *r* = 0.2453, *p* = 0.0507) (Figures 6(a) and 6(b)). This result suggested that the effect of the surround suppression on neuronal CS might not depend on surround suppression strength but was likely related to the mechanisms of surround suppression.

**Figure 7:**
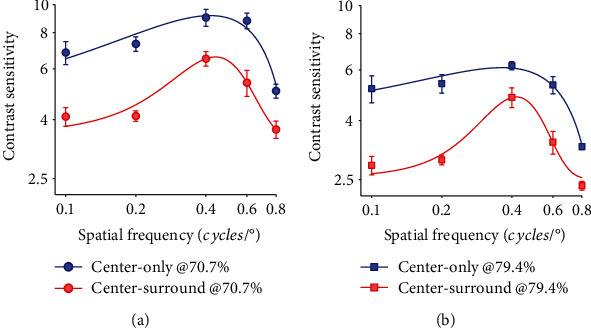
The contrast sensitivity versus stimulus spatial frequency (CSF) functions of NSS neurons (*n* = 61) measured at performance accuracy of 70.7% (a) and 79.4%, respectively, (b) with (red color) and without (blue color) surround stimuli. The blue and red solid curves were the best fits of CSFs with a Gauss function.

Because previous studies found that contrast sensitivity is correlated with stimulus SFs at both neuronal and perceptual level [27, 28, 30, 65], we thus compared the SS effects on the CS of NSS neurons with different preferred SFs. The neuronal CSF (CS vs. SF) functions at both performance criteria showed an inverted “U” shape with the CS maximizing at about 0.4 cpd but reducing toward lower and higher SFs (Figures 7(a) and 7(b)), which were basically similar to previous reports [28, 32, 51]. Two-way ANOVA showed that the surround suppression significantly reduced the CS of NSS neurons at both performance accuracies (70.7%: *F* (1,118) = 50.66, *p* = 0.0001; 79.4%: *F* (1,118) = 40.8, *p* = 0.0001), and the effect exhibited a significant dependence on the preferred SFs (70.7%: *F* (4,118) = 10.48, *p* = 0.0001; 79.4%: *F* (1,118) = 8.11, *p* = 0.0001). Further one-way ANOVA displayed that the reduction of CS in center-only condition versus center-surround condition varied significantly among groups of neurons with different SFs (70.7%: *F* (4,315) = 2.46, *p* = 0.0457; 79.4%: *F* (4,315) = 5, 21, *p* = 0.0004). Relative to center-only condition, the mean CS of neurons with preferred SF of 0.1, 0.2, 0.4, 0.6, and 0.8 in center-surround condition reduced by 27.03%, 30.56%, 18.15%, 20.07%, and 17.97% at 70.7% performance accuracy and 29.92%, 27.02%, 15.83%, 18.42%, and 18.11% at 79.4% performance accuracy, respectively.

### 3.3. Mechanisms of Surround Suppression Effect on Neuronal Contrast Sensitivity

**Figure 8:**
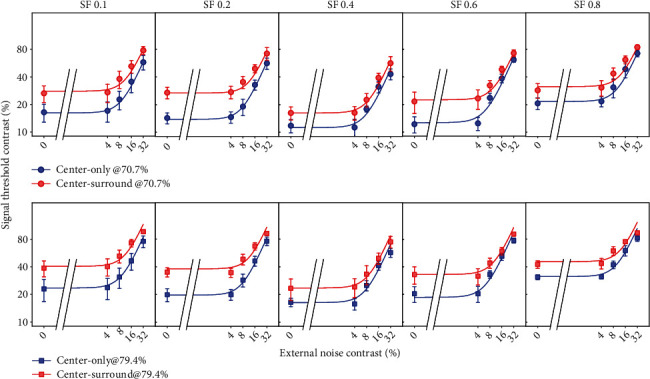
The log signal contrast threshold versus external noise contrast (TvC) functions at two performance criteria for NSS neurons (*n* = 61) with different preferred SFs. The filled circles and squares with error bars represent, respectively, the mean signal contrast threshold at 70.7% and 79.4% performance accuracy with (red color) and without (blue color) the presence of surround stimuli. The blue and red solid curves are the best-fitting TvC functions with PTM model under each condition.

To identify the mechanisms of surround suppression effect on the CS of different neuronal populations, we first compared the threshold contrast (TC) versus external noise contrast (TvC) functions from neurons with and without surround stimuli at different SFs (Figure 8). Three-way ANOVA (surround suppression × SF × external noise level) showed that the surround suppression significantly increased TC at two performance criteria (70.7%: *F* (1,640) = 182.776, *p* < 0.0001; 79.4%: *F* (1,640) = 272.74, *p* < 0.0001); the main effect displayed no interaction with external noise level (70.7%: *F* (4,640) = 1.432, *p* = 0.222; 79.4%: *F* (4,640) = 0.621, *p* = 0.648) but was significantly dependent on SF (70.7%: *F* (4,640) = 4.135, *p* = 0.003; 79.4%: *F* (4,640) = 5.508, *p* < 0.0001).

To quantify the SS effect on the TvC functions at the system level, we fitted TvCs of neuronal populations with and without surround stimuli at different preferred SFs using the perceptual template model (PTM) (Eq. (3)). This model had systematically characterized visual attention and perceptual learning effects in previous studies [38, 42, 44–46, 58]. By adjusting the coefficients *A*_*a*_, *A*_*f*_, and *A*_*m*_ in Eq. (3), we constructed a total of eight models, including one full model and seven reduced models, each of which represents a possible mechanism of surround suppression effect predicted by PTM. Our modeling analysis identified two different mechanisms of SS effect for neurons at lower (0.1 and 0.2 cpd) and higher (0.4-0.8 cpd) SF domains.

For all observers and their averages, the best fitting model to the TvC functions (*r*^2^ > 0.973) of neurons with preferred SF of 0.1 and 0.2 cpd identified a combined mechanism of SS effect (S-Figure 1 and S-Table 1): (1) stimulus suppression (equivalent to internal additive noise elevation), as indicated by *A*_*a*_ of 2.575 ± 1.997, 2.607 ± 1.453, and 2.521 ± 1.996 in SF 0.1, SF 0.2, and their averages and (2) increased external noise admission, as indicated by *A*_*f*_ of 1.479 ± 0.443, 1.327 ± 0.218, and 1.494 ± 0.509 in SF 0.1, SF 0.2, and their averages. This model (*A*_*a*_ and *A*_*f*_) was statistically identical to the full model (all *p* > 0.5) while all the other reduced models were statistically different from the full model (all *p* < 0.05). However, for all observers and their averages, the best fitting model to the TvC functions (r^2^> 0.92) for neurons with preferred SF of 0.4, 0.6, and 0.8 cpd showed that three models (*A*_*a*_, *A*_*a*_&*A*_*m*_, and *A*_*a*_&*A*_*f*_) were statistically equivalent to the full model (all *p* > 0.5) while the rest of the reduced models were significantly different from the full model (all *p* < 0.05) (S-Figure 1 and S-Table 1). This result suggested that SS effect on neuronal TvCs at SF 0.4, 0.6, and 0.8 cpd could be caused by the only mechanism of stimulus suppression (equivalent to internal additive noise elevation) indicated by the change of *A*_*a*_.

### 3.4. Neuronal Mechanism of Surround Suppression

**Figure 9:**
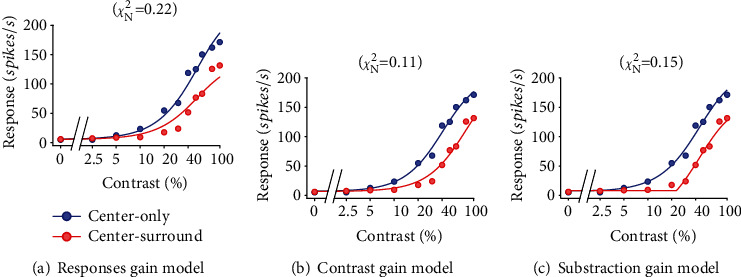
Responses and fits of a sample neuron to models accounting for surround suppression through different mechanisms. Red and blue circles and curves represent the contrast response functions and the model fitting curves with and without surround stimuli.

To further identify the neuronal mechanism of SF-dependent SS effect revealed by the PTM modeling, we fit the contrast response function of each NSS neuron with three models adapted from the Michaelis-Menten equation (see Material and Methods), including the response gain, contrast gain, and response subtraction model constructed by a previous study [2]. As shown by the fitting of a sample neuron (Figure 9), the response gain model accounts for SS effect through a divisive change in the neuron's response by a vertical scaling of the contrast response(Figure 9(a)); the contrast gain model accounts for influence effect through a horizontal shift of the contrast response function (Figure 9(b)); the subtraction model denotes the SS influence with a subtractive change of a uniform reduction of contrast response function (Figure 9(c)). This neuron is best characterized by the contrast gain model as indicated by the lowest *χ*_*N*_^2^ value.

**Figure 10:**
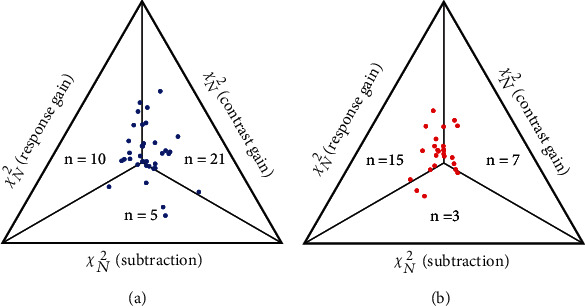
A three-way comparison of the three models for surround suppressive neurons with low preferred SFs (a) and high preferred SFs (b). Axis (lengths of sides of the triangle) units are normalized to make the total *χ*_*N*_^2^ of each model equal 1. The distance of a point from each bounding axis represents its *χ*_*N*_^2^ for the fit to that model. Because the lowest *χ*_*N*_^2^ value indicate the best fit, points near a bounding axis represent neurons for which model performed better than the others.

The fitting result of 61 NSS neurons, including 36 neurons with lower preferred SFs (0.1 and 0.2 cpd) and 25 neurons with higher preferred SFs (0.4-0.8 cpd), was illustrated by plotting the value of *χ*_*N*_^2^  from three models using an equilateral triangle with each side representing one of the three models (Figure 10). A point at the center of the triangle represented a neuron that was equally best fitted by all three models, and a point's varied distance to each side of the triangle was proportional to the *χ*_*N*_^2^ value of the corresponding model. The best fitting model for a neuron was represented by a point closest to the corresponding side of the triangle. Among 36 neurons in the low SF group, 10 neurons were best fitted by the response gain model, 21 neurons by the contrast gain model, and only 5 neurons by the subtraction model. Among 25 neurons in the high SF group, 15 neurons were best fitted by the response gain model, 7 neurons by the contrast gain model, and only 3 ones by the subtraction model. Therefore, SS effect on the contrast response function of neurons with low and high SFs was also varied: neurons with low SFs were mostly characterized by the contrast gain model, whereas neurons with higher SFs were chiefly characterized by the response gain model.

## 4. Discussion

### 4.1. Effect of Surround Suppression on the Contrast Sensitivity of V1 Neurons

Surround suppression (SS) is a phenomenon that a neuron's response to stimuli in the classic receptive field (cRF) is suppressed by a simultaneous stimulation in the region beyond cRF. For SS that has been widely reported in neurons across different hierarchical stages of visual information processing [9, 66, 67], it is commonly considered that SS plays a critical role in perceptual identification of objects from the background [8, 10, 68–71]. Although the characteristic of SS effects on neuronal response has been studied extensively [7, 9, 13, 33, 64, 72–76], the mechanisms that SS mediates visual information encoding in the cRF are still not fully understood [3, 22, 69, 77–83].

Luminance contrast detection is critical for perceptual identification of object's shape, size, and motion states [20, 21]. Psychophysical experiments report that contrast detection of contours in the central RF is impaired by contextual stimuli in the background [22, 84–86]. However, studies at the cellular level show that the SS effects on the response contrast sensitivity of neurons in the visual cortex are diverse. For example, some studies report that SS facilitates neuronal response to low-contrast visual stimuli but inhibit the response to high-contrast stimuli presented in the cRF [13, 77, 87]. Based on the contrast-response function fitting, some authors suggest that SS may modulate neuronal contrast sensitivity through different effects of response gain, contrast gain, and response subtraction [2, 23, 35]. Therefore, it is still unclear how SS modulates neuronal contrast encoding in the visual cortex. Previous studies have shown that perceptual contrast sensitivity is closely correlated with neuronal response contrast sensitivity [26–28, 30], and both of them depend on stimulus spatial frequencies (SFs) [24, 25, 27–32]. Moreover, a recent study report that the SS strength of V1 neurons is also positively correlated with their preferred SFs [33]. Therefore, it is speculated that SS effect on the response contrast sensitivity may vary among neuronal populations with different preferred SFs. We examined this possibility in the current study by comparing the SS effects on the contrast sensitivity between V1 neurons with different preferred SFs and found that SS had a larger effect on neurons with lower SF domain than those with higher one. Our results suggest that the SS influence on stimulus contrast encoding may depend on neuronal populations with different SFs. It is unknown if SS maps in the visual cortex have a correlation with the SF maps [88]; although, it has no evident relationship with the orientation maps [89]. Further studies are needed to clarify this issue.

### 4.2. Mechanisms of Surround Suppression

Although a considerable number of studies at the cellular level have shown that surround suppression (SS) may depend on multiple information encoding from feed-forward, lateral, and feedback connections [14, 66, 76, 80, 81, 90–93], the underlying mechanisms are not fully understood [3, 19]. For example, it is unclear whether SS results from an enhanced inhibition or a weakened excitation or both in the neural circuitry [3]. Some studies suggest that SS is primarily due to a reduction of cortical excitation [15, 94], which is consistent with the psychophysical experiment reporting that N-methyl d-aspartate receptor hypofunction reduces visual contextual integration [95]. However, other studies suggest that SS is caused by alterations of intracortical inhibition [16, 96–99], and still others show that SS can be affected by top-down influence through a disinhibition mechanism based on the interaction between different types of interneurons and excitatory pyramidal neurons [10, 18, 100]. The mechanisms of SS effect on neuronal contrast sensitivity are also diverse at the cellular level. Based on the contrast-response function fitting of individual neurons, SS may affect neuronal contrast sensitivity through response gain, contrast gain, and response subtraction [2, 23, 34, 35]. To reconcile these disagreements, it is necessary to analyze SS effects at the system level by treating the visual system as a perceptual template with signal input and perceptual output.

This study used the external noise paradigm and the perceptual template model (PTM) to examine the SS effect on the contrast threshold of V1 neurons with different preferred SFs. Based on PTM analysis of threshold versus external noise contrast (TvC) functions, we showed that surround suppression affected neuronal TvCs through different mechanisms: an increased internal additive noise and an enhanced impact of external noise for neurons with lower SFs (0.1-0.2 cpd), but only improved internal additive noise for neurons with higher SFs (0.4-0.8 cpd).

To further examine if the SF-dependence of SS effect occurred at the cellular level, we fitted the contrast response functions of NSS neurons with low and high SFs using three different models constructed previously [2, 23]. Our results showed that the SS effect on neuronal contrast response functions also displayed a variation between neurons with low and high SFs. The SS effect for neurons with low SFs was mediated dominantly through a reduced contrast gain, whereas the SS effect for neurons with high SFs was achieved dominantly through a lowered response gain.

Although the mechanism of SS effect on neuronal contrast sensitivity exhibited a dependence on neuronal preferred SFs both at the system (or perceptual template) level and the cellular level as revealed by PTM analysis and contrast-response function fitting, however, how these two types of mechanisms relate to each other is currently unclear. According to perceptual learning effects in visual contrast detection, some studies reported a combined mechanism of learning effect in stimulus enhancement (or equivalent internal additive noise reduction) and external noise exclusion at the perceptual template level [59, 101], whereas one physiological study found the learning effect in the contrast gain for neurons in the V1 cortex [51]. A similar mechanism is also observed in the visual attention effect as well as spatial frequency-dependent and individual-dependent effect on neuronal contrast sensitivity in the early visual areas [20, 30, 102]. Furthermore, our recent studies show that suppression of top-down influence decreases the neuronal contrast sensitivity in the V1 cortex. This top-down suppression may be mediated by a stronger effect of internal noise elevation than of external noise admission at the perceptual template level [48], whereas it may occur through a larger effect in the reduction of response gain than of contrast gain on the contrast-response function of V1 neurons [32], and this top-down influence in response gain may relate to alterations in excitatory glutamatergic neurotransmission [103–105]. Finally, recent studies suggest that increased GABAergic inhibition may underlie noise filtering in the visual signal perception [106, 107]. Taken together, it is possible that the network mechanism of surround suppression in the internal additive noise plus external noise exclusion observed for low-SF neurons in this study may involve a reduction of both excitation and inhibition in the local neural circuit, which chiefly causes a lowered contrast gain for neuronal contrast-response functions, whereas the single mechanism of surround suppression in the internal additive noise observed for high-SF neurons may relate predominantly to a reduction of excitation in the local neural circuit, which mainly results in a decreased response gain. We are designing a new experiment to examine this possibility by simultaneously observing changes at the perceptual template level and cellular level after modifying excitation-inhibition balance through administration of agonists or antagonists of glutamatergic and GABAergic receptors.

## 5. Conclusion

In conclusion, surround suppression significantly decreased the contrast sensitivity of V1 neurons, but the effect was mediated by varied mechanisms for different neuronal populations. For neurons with low preferred SFs, the surround suppression effect may occur by improving both internal additive noise and the impact of external noise at the system level, which may cause a larger reduction in neuronal contrast sensitivity through a lowered contrast gain. By contrast, for neurons with high preferred SFs, the surround suppression may be mediated only by increasing internal additive noise at the system level, which may result in a less reduction in neuronal contrast sensitivity through a decreased response gain.

## Figures and Tables

**Table 1:** RF properties of different types of neurons, including neurons with evident surround suppression (NSS), neurons with no surround suppression (NOS), and neurons with surround facilitation (NSF). Cell *N*, cRF, sRF, and SI denote neuronal number, center receptive field, surround receptive field, and surround suppression index, respectively.

Type	Cell *N*	cRF size [°]	sRF outer diameter [°]	sRF inner diameter [°]	SI
NSS	61	5.83 ± 1.48	12.28 ± 2.24	8.73 ± 2.24	0.21 ± 0.11
NOS	24	5.57 ± 1.32	NA	9.10 ± 1.00	NA
NSF	8	NA	NA	NA	NA

NA represents values that are not measureable.

## Data Availability

All data have been included in the manuscript, and supplementary information files are available from the corresponding author under reasonable requests.

## Supplementary Materials

Supplementary Materials In the Supplementary Material, S-Figure 1 shows the plots of nested PTM modeling based on preferred SFs. S-Table 1 shows the parameter estimates of the PTM fitting result.
