# Supplementary material for: Mechanisms of Surround Suppression Effect on the Contrast Sensitivity of V1 Neurons in Cats
Source: Neural Plast. 2022 Mar 8;2022:5677655. doi: 10.1155/2022/5677655 (PMC8923783; doi:10.1155/2022/5677655)
Supplement: Supplementary Materials — In the Supplementary Material, S-Figure 1 shows the plots of nested PTM modeling based on preferred SFs. S-Table 1 shows the parameter estimates of the PTM fitting result. [file 5677655.f1.pdf]

S- Figure 1

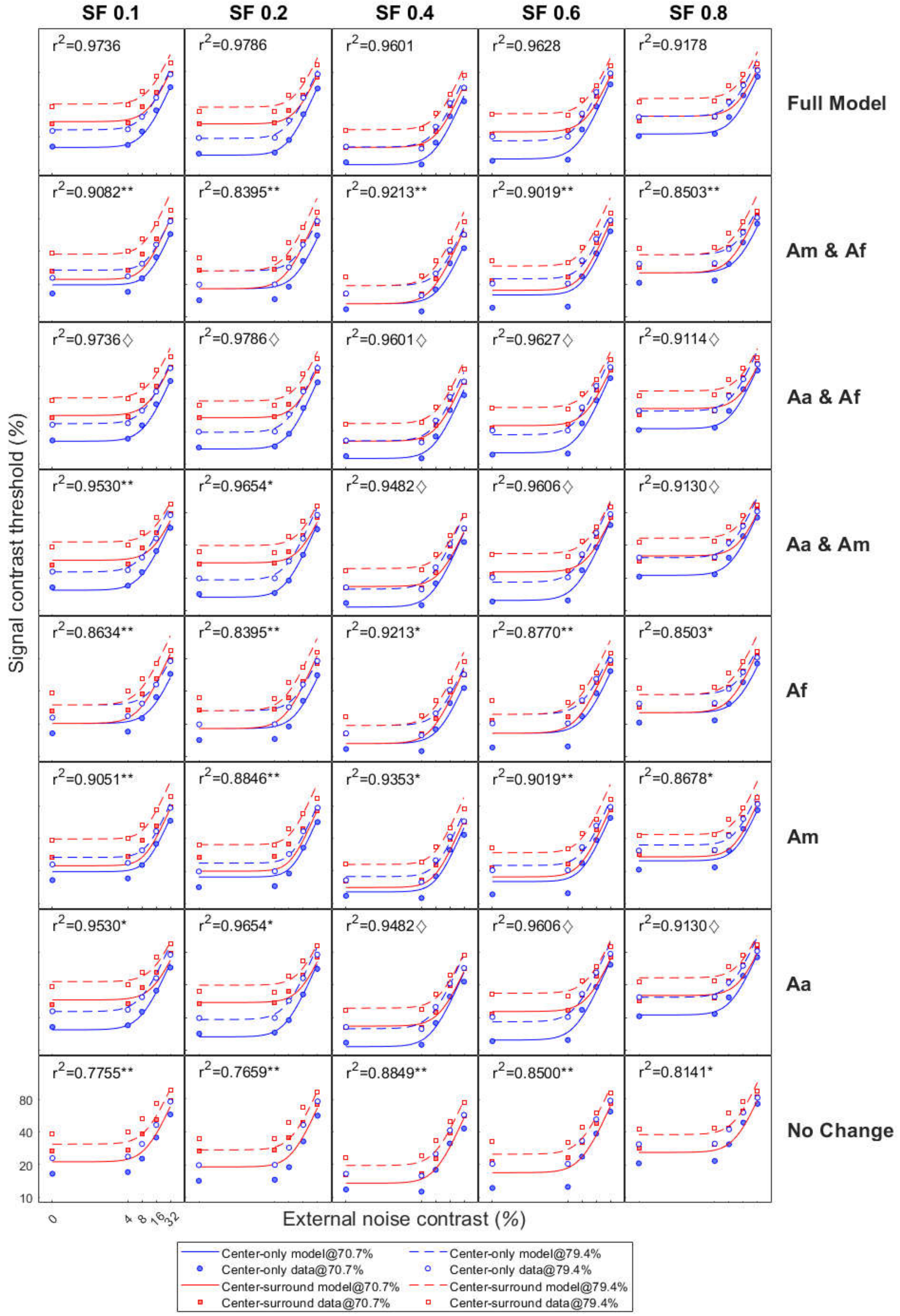

S-Figure 1. This figure matrix shows the results of PTM modeling arranged by SF (columns) and models (rows). In each panel, dots and squares represent mean contrast thresholds at each noise level, and smooth curves represent model fit to the data. Goodness-of-fit statistics for a given model are

provided, * and ** indicate significant difference between full model and each reduced model. ◊ represents statistically equivalence between full model and each reduced model.

S-Table 1: P vaules of model comparisons between neuronal TvC functions at different spatial frequencies (SFs) in center-only (cRF) and center-surround (cRF+sRF) condition. r^2 indicates goodness of fit. N_a, N_{ext}, N_m, β and γ denotes respectively the standard deviation of internal additive noise, the standard deviation of external noise, the proportional constant of multiplicative noise, the gain of the perceptual template, and the exponent of the non-linear transducer. A_a, parameter associated with signal enhancement or equivalent to internal additive noise reduction; A_m, parameter associated with the proportional constant of multiplicative noise; A_f, parameter associated with external noise exclusion.

Full Model

SF	SF 0.1		SF 0.2		SF 0.1-0.2		SF 0.4		SF 0.6		SF 0.8		SF 0.4-0.8	
Parameters	cRF	cRF+sRF	cRF	cRF+sRF	cRF	cRF+sRF	cRF	cRF+sRF	cRF	cRF+sRF	cRF	cRF+sRF	cRF	cRF+sRF
r^2	0.9736		0.9786		0.9736		0.9601		0.9628		0.9178		0.9591	
Na	0.0659±0.0678		0.0518±0.0400		0.0694±0.0772		0.0524±0.0468		0.0550±0.0430		0.0874±0.0482		0.0595±0.0441	
Nm	0.1393±0.1813		0.0801±0.1463		0.1399±0.1846		0.1055±0.1665		0.0696±0.1381		0.0257±0.0882		0.0635±0.1353	
β	0.6812±0.2266		0.6288±0.1163		0.6995±0.2974		0.7644±0.1721		0.5679±0.1123		0.5355±0.1522		0.6354±0.1134	
γ	1.6964±1.1733		1.4478±0.7947		1.7016±1.1791		1.5031±0.8423		1.2577±0.5990		1.1410±0.3356		1.2962±0.6301	
Aa	2.2828±1.7445		2.4038±1.1691		2.3461±1.8211		1.6630±0.8384		2.1336±1.4204		1.5370±0.3517		1.8184±0.8869	
Am	2.1617±1.9584		2.0547±1.8478		2.1352±1.9787		1.6708±1.4963		1.9432±1.7891		1.7203±1.4675		1.8592±1.6894	
Af	1.4306±0.4116		1.2907±0.2303		1.4578±0.5117		1.2254±0.2165		1.1409±0.1450		1.2109±0.2326		1.2062±0.1749	

Am & Af

SF	SF 0.1		SF 0.2		SF 0.1-0.2		SF 0.4		SF 0.6		SF 0.8		SF 0.4-0.8	
Parameters	cRF	cRF+sRF	cRF	cRF+sRF	cRF	cRF+sRF	cRF	cRF+sRF	cRF	cRF+sRF	cRF	cRF+sRF	cRF	cRF+sRF
r^2	0.9082		0.8395		0.9082		0.9213		0.9019		0.8503		0.8931	
p	0.0001		0		0.0001		0.0035		0.0005		0.0061		0.0005	
Na	0.0873±0.0927		0.0695±0.0603		0.0849±0.0857		0.0661±0.0561		0.0690±0.0471		0.1016±0.0526		0.0755±0.0534	
Nm	0.1193±0.1649		0.0996±0.1418		0.1252±0.1702		0.0801±0.1358		0.0748±0.1313		0.0380±0.0807		0.0545±0.1093	
β	0.7253±0.2718		0.6717±0.1407		0.7153±0.2493		0.7976±0.1997		0.6004±0.1068		0.5565±0.1192		0.6712±0.1211	
γ	1.7766±1.2783		1.5795±0.9472		1.7794±1.2375		1.5085±0.8977		1.2769±0.6041		1.1927±0.4503		1.2899±0.6046	
Aa	1		1		1		1		1		1		1	
Am	1	3.0548±3.3824	1	4.1488±3.9661	1	2.8785±3.2821	1	4.1408±3.9551	1	3.1006±3.5008	1	3.8048±3.8454	1	3.4562±3.7203
Af	1	1.6154±0.5703	1	1.4248±0.3408	1	1.5835±0.5055	1	1.2625±0.2612	1	1.2448±0.2039	1	1.3231±0.2751	1	1.3179±0.2459

Aa & Af

SF	SF 0.1		SF 0.2		SF 0.1-0.2		SF 0.4		SF 0.6		SF 0.8		SF 0.4-0.8	
Parameters	cRF	cRF+sRF	cRF	cRF+sRF	cRF	cRF+sRF	cRF	cRF+sRF	cRF	cRF+sRF	cRF	cRF+sRF	cRF	cRF+sRF

r^2	0.9736		0.9786		0.9736		0.9601		0.9627		0.9114		0.9644	
p	1		1		1		1		0.934		0.3329		1	
Na	0.0704±0.0695		0.0529±0.0385		0.0749±0.0755		0.0601±0.0526		0.0525±0.0405		0.0871±0.0419		0.0623±0.0445	
Nm	0.1324±0.1870		0.0974±0.1615		0.1308±0.1839		0.1001±0.1693		0.0875±0.1581		0.0342±0.0947		0.0691±0.1426	
β	0.7037±0.2371		0.6409±0.1035		0.7207±0.3020		0.7914±0.2109		0.5736±0.1139		0.5310±0.1028		0.6464±0.1219	
γ	1.4538±0.7982		1.3282±0.4782		1.4623±0.8641		1.4034±0.7500		1.2310±0.4723		1.1268±0.2646		1.2256±0.4890	
Aa	1	2.5748±1.9971	1	2.6074±1.4531	1	2.5212±1.9760	1	1.6888±0.8660	1	2.2839±1.6314	1	1.5416±0.3817	1	1.9119±1.1795
Am	1		1		1		1		1		1		1	
Af	1	1.4792±0.4434	1	1.3270±0.2176	1	1.4944±0.5087	1	1.2571±0.2273	1	1.1454±0.1362	1	1.2056±0.1866	1	1.2251±0.1689

Aa & Am

SF	SF 0.1		SF 0.2		SF 0.1-0.2		SF 0.4		SF 0.6		SF 0.8		SF 0.4-0.8	
Parameters	cRF	cRF+sRF	cRF	cRF+sRF	cRF	cRF+sRF	cRF	cRF+sRF	cRF	cRF+sRF	cRF	cRF+sRF	cRF	cRF+sRF
r^2	0.953		0.9654		0.953		0.9482		0.9606		0.913		0.9592	
p	0.0071		0.014		0.0071		0.0712		0.401		0.4022		1	
Na	0.0624±0.0675		0.0496±0.0390		0.0609±0.0676		0.0496±0.0439		0.0492±0.0381		0.0789±0.0387		0.0520±0.0391	

Nm	0.1301±0.1736		0.0909±0.1486		0.1288±0.1733		0.0844±0.1373		0.0749±0.1399		0.0296±0.0888		0.0615±0.1266	
β	0.6285±0.2490		0.5898±0.1005		0.6119±0.2523		0.7035±0.1586		0.5403±0.1102		0.4952±0.0799		0.5884±0.1035	
γ	1.6139±1.0406		1.4598±0.8321		1.6577±1.0698		1.4932±0.8645		1.2922±0.6124		1.1457±0.3220		1.3243±0.6567	
Aa	1	2.5769±2.0106	1	2.6052±1.4425	1	2.6363±2.0247	1	1.6347±0.8025	1	2.2851±1.5047	1	1.5935±0.3871	1	2.0502±1.2281
Am	1	2.5802±2.9271	1	2.7441±3.0424	1	2.5341±2.8169	1	3.6856±3.7130	1	2.0745±2.4176	1	2.5239±2.5406	1	2.3627±2.7912
Af	1		1		1		1		1		1		1	

Af

SF	SF 0.1		SF 0.2		SF 0.1-0.2		SF 0.4		SF 0.6		SF 0.8		SF 0.4-0.8	
Parameters	cRF	cRF+sRF	cRF	cRF+sRF	cRF	cRF+sRF	cRF	cRF+sRF	cRF	cRF+sRF	cRF	cRF+sRF	cRF	cRF+sRF
r^2	0.8634		0.8395		0.8634		0.9213		0.877		0.8503		0.8931	
p	0		0		0		0.0122		0.0004		0.0203		0.0019	
Na	0.1101±0.1008		0.1028±0.0615		0.1193±0.1011		0.0819±0.0628		0.0820±0.0492		0.1160±0.0520		0.0896±0.0548	
Nm	0.0905±0.1706		0.0414±0.1171		0.0838±0.1636		0.0700±0.1517		0.0534±0.1341		0.0258±0.0985		0.0445±0.1216	
β	0.7844±0.3927		0.7344±0.1663		0.7997±0.3708		0.8427±0.2201		0.6206±0.1165		0.5752±0.1127		0.6991±0.1319	
γ	1.5370±1.0992		1.1780±0.4493		1.3886±0.9471		1.3281±0.7026		1.1569±0.4789		1.1332±0.4300		1.1794±0.5001	

Aa	1		1		1		1		1		1		1	
Am	1		1		1		1		1		1		1	
Af	1	1.8572±0.6304	1	1.7157±0.3174	1	1.8807±0.6251	1	1.4074±0.2698	1	1.3546±0.1550	1	1.4305±0.2314	1	1.4471±0.2173

Am

SF	SF 0.1		SF 0.2		SF 0.1-0.2		SF 0.4		SF 0.6		SF 0.8		SF 0.4-0.8	
Parameters	cRF	cRF+sRF	cRF	cRF+sRF	cRF	cRF+sRF	cRF	cRF+sRF	cRF	cRF+sRF	cRF	cRF+sRF	cRF	cRF+sRF
r^2	0.9051		0.8846		0.9051		0.9353		0.9019		0.8678		0.9161	
p	0.0002		0		0.0002		0.0435		0.0018		0.0457		0.0094	
Na	0.0526±0.0552		0.0427±0.0363		0.0512±0.0528		0.0469±0.0439		0.0523±0.0381		0.0765±0.0374		0.0526±0.0394	
Nm	0.1293±0.1522		0.0815±0.1055		0.1354±0.1523		0.0821±0.1223		0.0747±0.1047		0.0450±0.0710		0.0642±0.0893	
β	0.6026±0.1645		0.5903±0.0892		0.6000±0.1647		0.7185±0.1766		0.5640±0.1208		0.4953±0.0648		0.6046±0.0941	
γ	1.8134±1.0826		1.5881±0.6379		1.7867±1.0455		1.5715±0.7996		1.3490±0.5650		1.2825±0.5089		1.3960±0.6088	
Aa	1		1		1		1		1		1		1	
Am	1	7.1970±3.9105	1	8.9123±2.7686	1	7.1641±3.9029	1	7.6149±3.7850	1	8.5164±3.2092	1	7.5166±3.9173	1	8.9048±2.8231
Af	1		1		1		1		1		1		1	

Aa

SF	SF 0.1		SF 0.2		SF 0.1-0.2		SF 0.4		SF 0.6		SF 0.8		SF 0.4-0.8	
Parameters	cRF	cRF+sRF	cRF	cRF+sRF	cRF	cRF+sRF	cRF	cRF+sRF	cRF	cRF+sRF	cRF	cRF+sRF	cRF	cRF+sRF
r^2	0.953		0.9654		0.953		0.9482		0.9606		0.913		0.9577	
p	0.0234		0.0437		0.0234		0.1845		0.6933		0.6948		0.8114	
Na	0.0678±0.0619		0.0564±0.0380		0.0668±0.0610		0.0528±0.0444		0.0512±0.0376		0.0793±0.0389		0.0585±0.0390	
Nm	0.0975±0.1682		0.0594±0.1381		0.0971±0.1718		0.0840±0.1651		0.0651±0.1440		0.0260±0.0957		0.0440±0.1215	
β	0.6218±0.1883		0.5962±0.0991		0.6170±0.1770		0.7116±0.1775		0.5391±0.0925		0.4946±0.0903		0.5952±0.0911	
γ	1.3779±0.7484		1.2391±0.4645		1.3956±0.7513		1.4011±0.7361		1.2119±0.4869		1.1500±0.3783		1.1935±0.4753	
Aa	1	3.0189±2.3942	1	2.7669±1.6327	1	3.0116±2.4616	1	1.8180±1.1528	1	2.3732±1.6573	1	1.6496±0.6426	1	2.0529±1.2421
Am	1		1		1		1		1		1		1	
Af	1		1		1		1		1		1		1	

No Change

SF	SF 0.1		SF 0.2		SF 0.1-0.2		SF 0.4		SF 0.6		SF 0.8		SF 0.4-0.8	
Parameters	cRF	cRF+sRF	cRF	cRF+sRF	cRF	cRF+sRF	cRF	cRF+sRF	cRF	cRF+sRF	cRF	cRF+sRF	cRF	cRF+sRF

r^2	0.7755	0.7659	0.7755	0.8849	0.85	0.8141	0.8555
p	0	0	0	0.0026	0.0003	0.0119	0.0007
Na	0.0745±0.0672	0.0664±0.0457	0.0769±0.0668	0.0668±0.0539	0.0669±0.0445	0.0942±0.0417	0.0719±0.0459
Nm	0.1084±0.1839	0.0696±0.1539	0.1187±0.1921	0.0780±0.1588	0.0628±0.1455	0.0235±0.0941	0.0570±0.1394
β	0.5769±0.1898	0.5528±0.0900	0.5841±0.1774	0.7036±0.1751	0.5314±0.1106	0.4815±0.0826	0.5823±0.0966
γ	1.5382±0.9457	1.3160±0.5392	1.5183±0.9813	1.3690±0.7465	1.2320±0.5740	1.1369±0.3298	1.2094±0.5108
Aa	1	1	1	1	1	1	1
Am	1	1	1	1	1	1	1
Af	1	1	1	1	1	1	1
